# Tryptophan metabolism enzymes are potential targets in ovarian clear cell carcinoma

**DOI:** 10.1002/cam4.6778

**Published:** 2023-12-07

**Authors:** Sumei Zhang, Yike Gao, Pan Wang, Shu Wang, Yuming Wang, Mei Li, Anqi Wang, Kun Zhao, Zixin Zhang, Jian Sun, Dan Guo, Zhiyong Liang

**Affiliations:** ^1^ Clinical Biobank, Peking Union Medical College Hospital Chinese Academy of Medical Sciences & Peking Union Medical College Beijing China; ^2^ Department of Medical Research Centre, Peking Union Medical College Hospital Chinese Academy of Medical Sciences & Peking Union Medical College Beijing China; ^3^ Department of Pathology, Molecular Pathology Research Centre, Peking Union Medical College Hospital Chinese Academy of Medical Science & Peking Union Medical College Beijing China; ^4^ Department of Pathology Affiliated Hospital of Hebei University Baoding Hebei Province China; ^5^ Department of Obstetrics and Gynaecology, Peking Union Medical College Hospital (PUMCH) Chinese Academy of Medical Sciences & Peking Union Medical College Beijing China; ^6^ National Clinical Research Centre for Obstetric & Gynaecologic Diseases Beijing China

**Keywords:** immunohistochemistry, ovarian clear cell carcinoma, tryptophan metabolism

## Abstract

**Aim:**

As the second most prevalent subtype of epithelial ovarian cancers, ovarian clear cell carcinoma (OCCC) is known for its chemoresistance to conventional platinum‐based therapy. In this work, we examined the tryptophan (Trp) metabolism enzymes' differential expression in patients with OCCC to assess the potential for personalised treatment.

**Methods:**

A total of 127 OCCC tissues were used to construct tissue microarrays, and immunohistochemistry (IHC) staining of the Trp enzymes IDO1, IDO2, TDO2 and IL4I1 was performed. The correlations between Trp enzyme expression and clinical characteristics were analysed.

**Results:**

Positive IDO1, IDO2, TDO2 and IL4I1 staining was identified in 26.8%, 94.5%, 75.6% and 82.7% of OCCC respectively. IDO1‐positive samples were more common in the chemoresistant group than in the platinum‐sensitive group (46.7% vs. 19.8%). Moreover, positive expression of IDO1, TDO2 and IL4I1 was related to advanced stage, metastasis, bilateral tumours, endometriosis and tumour rupture (*p* < 0.05) respectively. Univariate analysis revealed a significant association between bilateral tumours, lymph node metastasis, advanced stage, distant metastasis and aberrant cytology with a poor prognosis for OCCC, while the absence of residual tumour was correlated with a favourable outcome (*p* < 0.05). However, only bilateral tumours and lymph node metastases were related to a poor prognosis after multivariate analysis.

**Conclusion:**

This is the first study to investigate the expression of the Trp enzymes IDO1, IDO2, TDO2 and IL4I1 in OCCC tissues. IDO2, TDO2 and IL4I1 were detected in the majority of OCCC. Clinical traits were correlated with IDO1, IDO2, TDO2 and IL4I1 expression. IDO1 may be used as a therapeutic target given the large percentage of chemoresistant cases with IDO1 expression. These results will aid the development of personalised therapies for OCCC.

## 1 INTRODUCTION

Ovarian cancer is the most common gynaecological cancer, with a total of 198,412 deaths in 2019.^1^ Epithelial ovarian cancer can be classified into high‐grade serous ovarian carcinoma (HGSOC), low‐grade serous ovarian carcinoma (LGSOC), mucinous carcinoma (MC), ovarian clear cell carcinoma (OCCC), endometrioid carcinoma (EC) and other types based on histopathology.^2^ Compared with HGSOC (70%), OCCC accounts for only 10% of epithelial ovary cancers^3^ and has a poor prognosis.^4^ Platinum resistance is known to be the primary cause of the unsatisfactory outcomes of OCCC.^5^, ^6^, ^7^, ^8^

From a molecular standpoint, the increased antioxidant capacity of the cancer cells and the surrounding microenvironment was the primary cause of chemoresistance in ovarian cancer cells.^9^, ^10^ NQO1 is an antioxidant enzyme that takes part in ovarian cancer onset and progression.^10^ Its modulator, Nrf2 mediates antioxidant pathway and serves as a resistance mechanism in proteasome inhibitor‐resistant cells.^11^ Moreover, suppression of the NF‐κB signalling pathway, a key factor involved in innate immune response and inflammation, led to apoptosis and reduced cell proliferation in drug‐resistant ovarian cancer cells.^9^ For chemoresistant OCCCs, immunotherapy directed at the tumour microenvironment may therefore be the best option.

New immunotherapy strategies for the treatment of ovarian cancer are emerging and have the potential to prolong the survival of some patients.^12^ However, immunotherapy efficacy may be hindered by the immunosuppressive tumour microenvironment (TME) induced by tumour‐mediated metabolism in the host.^5^, ^13^, ^14^, ^15^, ^16^, ^17^ For example, tryptophan (Trp) is an essential amino acid affecting three major downstream pathways: the serotonin, indoleacetic and kynurenine (Kyn) pathways.^18^ The Kyn pathway has been shown to play an immunosuppressive role in various cancers.^4^, ^19^, ^20^, ^21^, ^22^, ^23^, ^24^, ^25^, ^26^ Multiple mechanisms of immunosuppression may be mediated by the Trp‐Kyn‐aryl hydrocarbon receptor (AhR) pathway, including depletion of Trp, immunosuppression directly induced by Kyn and Kyn‐bound AhR activity.^21^, ^22^, ^25^, ^26^, ^27^ Indoleamine‐2,3‐dioxygenase 1 and 2 (IDO1/2), tryptophan‐2,3‐dioxygenase (TDO2)^28^ and interleukin‐4‐induced‐1 (IL4I1)^22^ all participate in the process of Trp degradation to Kyn.

The enzyme IDO1 has been reported to be a therapeutic target in cancers, with evidence from pharmacological and genetic studies.^29^ Interestingly, the function of IDO1 in HGSOC is controversial. One study indicated that high IDO1 predicted longer survival and less sensitivity to carboplatin in HGSOC patients.^30^ Another study reported that HGSOC tumours with a high level of IDO1 might be resistant to therapy and were associated with significantly lower overall survival and progression‐free survival.^31^ IDO1 can catabolise Trp and suppress the proliferation of CD8+ effector T cells, natural killer cells and myeloid‐derived suppressor cells (MDSCs). In line with this, high IDO expression in ovarian cancer cells was found to be correlated with lower levels of tumour‐infiltrating lymphocytes, advanced surgical stage and decreased survival.^13^ Similarly, TDO2 catabolises the degradation of Trp to Kyn and is upregulated in ovarian cancer tissues compared with normal ovarian tissues, promoting the proliferation, migration and invasion of ovarian cancer cells.^32^ Recently, overexpression of IL4I1 was shown to activate AHR and promote tumour progression.^22^ Furthermore, elevated metabolism of l‐phenylalanine and l‐tyrosine by IL4I1 in ascites was associated with advanced disease stage, suggesting a role of IL4I1 in HGSOC progression.^33^

To date, the function of Trp metabolism in OCCC remains unclear. Therefore, in the present study, we investigated the in situ expression of enzymes of Trp metabolism in OCCC, including IDO1, IDO2, TDO2 and IL4I1, using immunohistochemistry (IHC) analysis of tissue microarrays (TMAs). This study aimed to explore the roles of Trp metabolism in OCCC.

## 2 MATERIALS AND METHODS

### 2.1 Patients and tumour specimens

This study recruited 127 patients diagnosed with OCCC. All patients received surgical treatment at our hospital between 2019 and 2022. The patients' diagnoses were confirmed by two experienced pathologists blinded to the first diagnosis based on the WHO guidelines introduced in 2020. Clinical and demographic data were collected and analysed. Tumour size was measured based on the longest axis of the primary tumour, and the larger tumour was considered if bilateral tumours existed. Abnormal cytology referred to malignant cells in ascites or peritoneal washings. Chemotherapy mainly included conventional platinum‐based therapy after surgery. Chemoresistance or platinum resistance was identified as recurrence within 6 months after completion of platinum‐based therapy.

Formalin‐fixed, paraffin‐embedded tissues were used to generate the TMA for IHC staining as previously described.^34^ The TMA was constructed by an expert pathologist from our hospital. Briefly, representative 1.8 mm tissue cores from the tumour area were selected for the purpose of constructing TMA blocks. Routine 4 μm sections were cut from the TMA blocks and stained with haematoxylin & eosin (H&E) to confirm the tumour purity and pathologic parameters.

This study was approved by the Institutional Review Board of Our Hospital (I‐22PJ112). Informed consent was obtained from all patients.

### 2.2 IHC staining

IHC staining was performed on 4‐μm paraffin sections using the DAKO Autostainer Link 48. The tissue epitopes were repaired using the automated water bath heating process with Dako PT Link (Dako, Glostrup, Denmark). The sections were incubated in TRIS‐EDTA retrieval solution (10 mM Tris, 1 mM EDTA pH 9.0) or citric acid buffer (10 mM, pH 6.0) at 98°C for 20 min. Then, they were subsequently incubated for 20 min with primary antibodies, followed by incubation with anti‐rabbit immuno‐peroxidase polymer (Envision FLEX/HRP) for 20 min. Primary antibodies included antibodies against IDO1 (EPR20374, Abcam, ab211017, dilution 1:4000), IDO2 (8322548, Proteintech, 25053‐1‐AP, dilution 1:100), TDO2 (4071714, Proteintech, 15880‐1‐AP, dilution 1:100) and IL4I1 (EPR22070, Abcam, ab222102, dilution 1:2000). The colour was developed with DAB substrate‐chromogen solution for 10 min. Finally, the sections were counterstained with haematoxylin.

### 2.3 Evaluation of IHC results

Positive IDO1, IDO2 and TDO2 staining was diffuse in the cytoplasm, while positive IL4I1 staining was granular and scattered in the cytoplasm.^22^, ^35^, ^36^, ^37^, ^38^, ^39^ The IHC controls are listed in Table S1.

Composite scores for IDO1, IDO2, TDO2 and IL4I1 were determined by two experienced pathologists based on both the intensity of staining and proportion of positive cells. The intensity score was determined on a scale of 0–3 (0—negative, 1—weak, 2—moderate, 3—intense staining), and the representative images for different intensity were shown in Figure S1. In addition, the percentage of positive cells was scored on a scale of 1–4 (1: 0%–24%, 2: 25%–49%, 3: 50%–74%, 4: 75%–100%). Sections with composite scores (the product of the intensity score and the percentage score) greater than or equal to 4 points were considered positive.

### 2.4 Statistical analysis

Statistical analyses of IDO1, IDO2, TDO2 and IL4I1 expression determined by IHC and clinical characteristics were carried out using nonparametric statistics. Pearson's coefficient was calculated to evaluate the relations between the expression of IDO1, IDO2, TDO2 and IL4I1 and clinical characteristics. Univariate and multivariate statistical analyses were applied to identify factors related to patient prognosis. The data were analysed with SPSS version 24.0 for Windows (SPSS Inc., Chicago, IL, USA). A value of *p* < 0.05 was considered statistically significant.

## 3 RESULTS

### 3.1 Demographic and clinical data of patients with OCCC

A total of 127 tissue samples from patients with OCCC were analysed in the present study. The clinicopathological characteristics of the OCCC patients are summarised in Table 1. The average age of the OCCC patients was 51 years, and 95 (74.8%) patients had early‐stage disease (Stage I & II). Additionally, bilateral tumours were detected in 17 (13.4%) patients. The mean value of the longest axis of the primary tumour was 12.1 cm. Thirteen patients were diagnosed with lymph node metastasis, and 11 were diagnosed with distant metastasis. Although 46 (36.2%) tumours had ruptured, the majority of patients did not have residual tumours. A history of endometriosis was reported in 86 (67.7%) women, and 123 (96.9%) patients received chemotherapy after surgery. The total number of patients who experienced chemotherapy resistance and recurrence was 15 (15.6%) and 23 (18.1%) respectively. During the follow‐up period, two patients died, and the progression‐free survival and overall survival were 16 and 18 months respectively. According to the IDO1, IDO2, TDO2 and IL4I1 IHC results, 34 (26.8%), 120 (94.5%), 96 (75.6%) and 105 (82.7%) patients had positive IHC staining respectively.

**TABLE 1:** Clinicopathological data of patients with ovarian clear cell carcinoma (*n* = 127).

Age (mean ± SD)	51 ± 10
Stage	
I	71(55.9%)
II	24 (18.9%)
III	25 (19.7%)
IV	7 (5.5%)
Bilateral tumours	17 (13.4%)
Tumour size/cm^a^	12.1 ± 4.7
Lymph node metastasis	13 (10.2%)
Distant metastasis	11 (8.7%)
Abnormal cytology^b^	33 (26.0%)
Tumour rupture	46 (36.2%)
No residual tumour/R0	119 (93.7%)
Endometriosis	86 (67.7%)
Thrombosis	25 (19.7%)
Chemotherapy	123 (96.9%)
Other therapy	27 (21.3%)
Recurrence	23 (18.1%)
Chemoresistance (*n* = 96)^c^	15 (15.6%)
Death	2 (1.6%)
PFS/month (mean ± SD)	16 ± 11
OS/month (mean ± SD)	18 ± 12
IDO1	34 (26.8%)
IDO2	120 (94.5%)
TDO2	96 (75.6%)
IL4I1	105 (82.7%)

^a^ Tumour size was the measurement of the longest axis of the primary tumour.

^b^ Abnormal cytology meant malignant cells in ascites or peritoneal washings.

^c^ Data deficiency due to lack of medical records.

### 3.2 Representative images of morphology and IHC results

**FIGURE 1:**
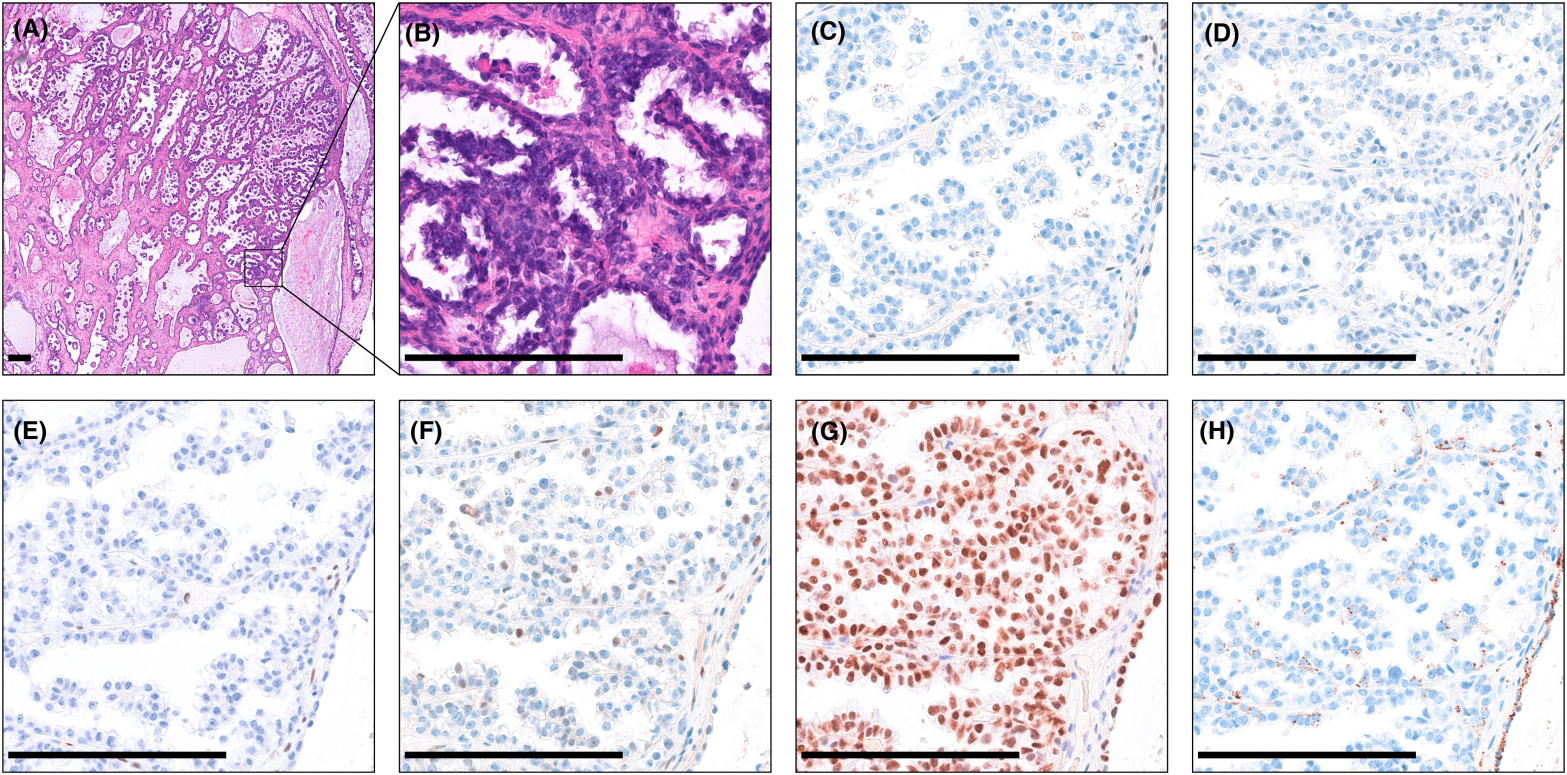
Typical histopathological and immunohistochemical images of OCCC. (A) Haematoxylin and eosin (H&E) figure of OCCC at low power (100×). (B) H&E figure of OCCC at high power (400×). (C–H). Immunohistochemical results of OCCC (400×). OCCC does not express ER (C), PR (D) or WT‐1 (E). It shows a wild‐type immunohistochemical staining pattern of P53 (F). Staining results of HNF1B (G) and Napsin A (H) are positive in OCCC. The scale bar is 200 μm.

**FIGURE 2:**
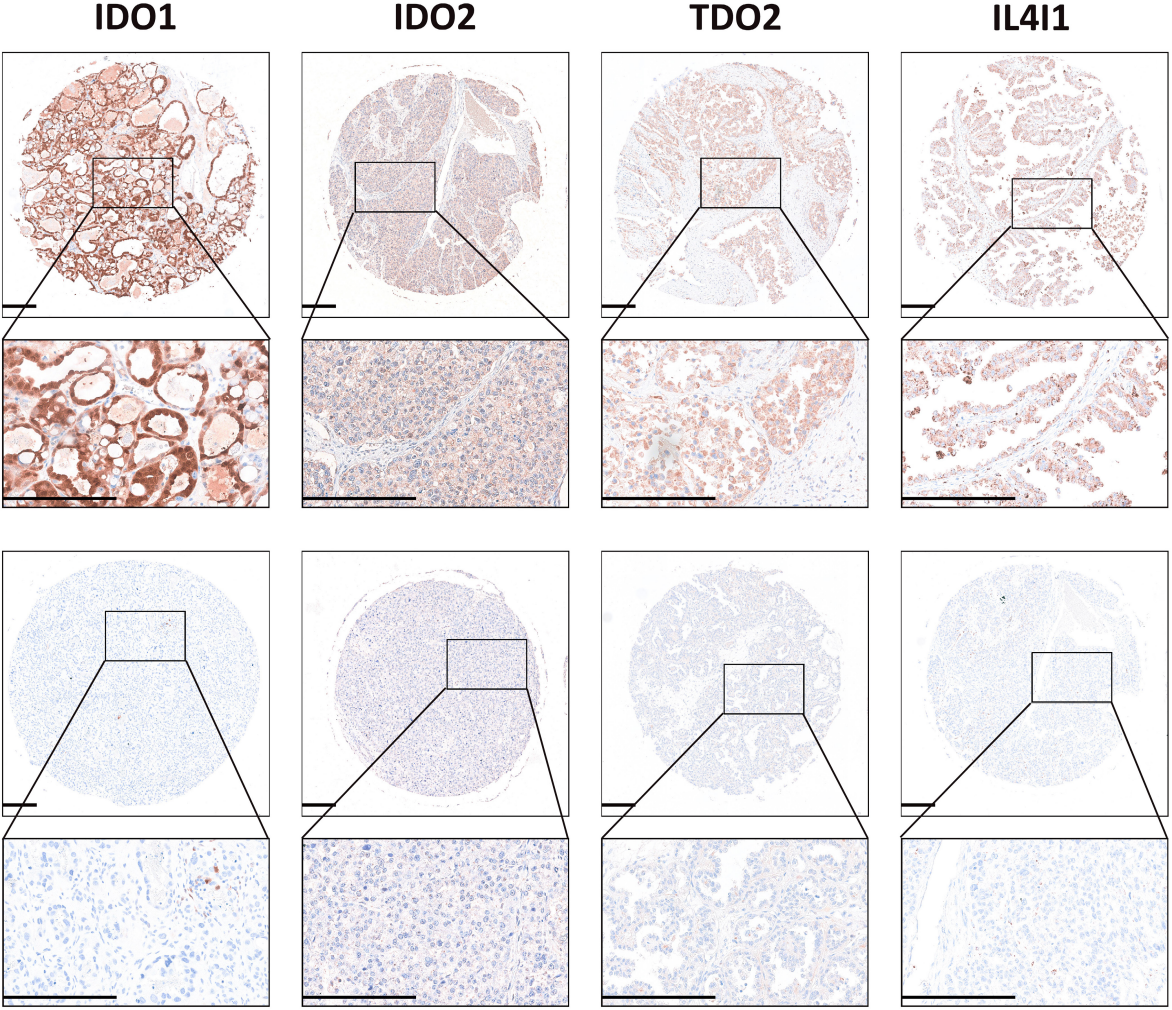
Positive or negative staining results of four tryptophan metabolic enzymes. The scale bar is 250 μm.

H&E sections showed typical morphology of OCCC with clear cells displaying tubulocystic or papillary architecture, as seen in Figure 1A,B. OCCC samples did not express ER (Figure 1C), PR (Figure 1D), WT‐1 (Figure 1E) based on IHC staining. More importantly, the positive HNF1B (Figure 1G) and Napsin A (Figure 1H) staining distinguished OCCC from other types of ovarian cancers.^40^ Representative examples of the expression of four Trp metabolism enzymes (IDO1, IDO2, TDO2 and IL4I1) in OCCC were shown in Figure 2.

### 3.3 Correlation between IDO1, IDO2, TDO2 and IL4I1 expression and clinical information

Interestingly, IDO1 expression was much lower than that of the other three genes in OCCC tissues (Table 1). We investigated the interrelationships of IDO1, IDO2, TDO2 and IL4I1 expression and the relationship of IDO1, IDO2, TDO2 and IL4I1 expression with clinical features; the results are shown in Table 2 and Table 3 respectively. As shown in Table 2, IDO1‐positive cases were more likely to express IL4I1 (*p* = 0.039), while TDO2 was often co‐expressed with IL4I1 (*p* = 0.011). Double‐positive staining of IDO1 and IL4I1 was detected in 32 samples (94.1% of IDO1‐positive samples). Among 96 TDO2‐positive samples, 84 (87.5%) exhibited simultaneous IL4I1 expression, while 21 tissues with TDO2‐negative staining were IL4I1 positive.

**TABLE 2:** Relations between expression of different Trp catabolising enzymes.

	Pos	IDO1	*p* value	Pos	IDO2	*p* value	Pos	TDO2	*p* value
		Neg			Neg			Neg	
IDO2	32 (94.1%)	88 (94.6%)	1.000	/	/	/	/	/	/
TDO2	29 (85.3%)	67 (72.0%)	0.124	93 (77.5%)	3 (42.9%)	0.105	/	/	/
IL4I1	32 (94.1%)	73 (78.5%)	**0.039** *	100 (83.3%)	5 (71.4%)	0.768	84 (87.5%)	21 (67.7%)	**0.011** *

* *p* < 0.05 as significance.

Abbreviations: Neg, negative; Pos, positive.

**TABLE 3:** Relations between expression of Trp catabolising enzymes and clinical information.

	Pos	IDO1	*p* value	Pos	IDO2	*p* value	Pos	TDO2	*p* value	Pos	IL4I1	*p* value
		Neg			Neg			Neg			Neg	
Late stage (III/IV)	13 (38.2%)	19 (20.4%)	**0.041** *	30 (25.0%)	2 (28.6%)	1.000	21 (21.9%)	11 (35.5%)	0.129	23 (21.9%)	9 (40.9%)	0.062
Bilateral tumours	6 (17.6%)	11 (11.8%)	0.577	16 (13.3%)	1 (14.3%)	1.000	8 (8.3%)	9 (29.0%)	**0.008** *	13 (12.4%)	4 (18.2%)	0.702
Lymph node metastasis	7 (23.3%)	6 (6.9%)	**0.033** *	12 (10.9%)	1 (14.3%)	0.572	8 (9.0%)	5 (17.9%)	0.338	10 (10.5%)	3 (13.6%)	0.967
Distant metastasis	6 (16.7%)	5 (5.4%)	0.069	10 (8.3%)	1 (14.3%)	0.478	7 (7.3%)	4 (12.9%)	0.549	10 (9.5%)	1 (4.5%)	0.735
Abnormal cytology	9 (26.5%)	24 (25.8%)	0.940	31 (25.8%)	2 (28.6%)	1.000	23 (24.0%)	10 (32.3%)	0.360	28 (26.7%)	5 (22.7%)	0.702
Tumour rupture	10 (29.4%)	36 (38.7%)	0.334	42 (35.0%)	4 (57.1%)	0.435	34 (35.4%)	12 (38.7%)	0.740	34 (32.4%)	12 (54.5%)	**0.049** *
Endometriosis	20 (58.8%)	66 (71.0%)	0.195	82 (68.3%)	4 (57.1%)	0.842	70 (72.9%)	16 (51.6%)	**0.027** *	68 (64.8%)	18 (81.8%)	0.120
Thrombosis	7 (20.6%)	18 (19.4%)	0.877	22 (18.3%)	3 (42.9%)	0.273	17 (17.7%)	8 (25.8%)	0.324	20 (19.0%)	5 (22.7%)	0.920
Recurrence	8 (23.5%)	15 (16.1%)	0.338	23 (19.2%)	0 (0)	0.438	15 (15.6%)	8 (25.8%)	0.201	18 (17.1%)	5 (22.7%)	0.754
Chemoresistance (*n* = 96)^a^	7 (30.4%)	8 (11.0%)	0.056	15 (16.9%)	0 (0)	0.521	10 (14.7%)	5 (17.9%)	0.938	12 (15.4%)	3 (16.7%)	1.000
Death	1 (2.9%)	1 (1.1%)	0.465	2 (1.7%)	0 (0)	1.000	1 (1.0%)	1 (3.2%)	0.430	2 (1.9%)	0 (0)	1.000

* *p* < 0.05 as significance.

Abbreviations: Neg, negative; Pos, positive.

^a^ Data deficiency due to lack of medical records.

Furthermore, we analysed the relationship between IDO1, IDO2, TDO2 and IL4I1 expression and some clinical features, such as advanced stage, bilateral ovarian carcinoma, lymph node metastasis, distant organ metastasis, abnormal cytology, tumour rupture, complications (endometriosis or thrombosis), cancer recurrence, chemotherapy drug resistance and death. As shown in Table 3, IDO1 expression was related to advanced stage (*p* = 0.041) as well as lymph node metastasis (*p* = 0.033). Negative TDO2 expression was significantly correlated with bilateral tumours (29% vs. 8.3%, *p* < 0.01) and endometriosis (51.6% vs. 72.9%, *p* < 0.05). Similarly, tumour rupture was observed to be related to negative IL4I1 expression (54.5% vs. 32.4%, *p* < 0.05).

### 3.4 Factors related to OCCC prognosis

We analysed the influence of IDO1, IDO2, TDO2 and IL4I1 expression and clinical characteristics on OCCC prognosis, and the results are shown in Table 4. In the univariate statistical analysis, there was no significant correlation of progression‐free survival with IDO1, IDO2, TDO2 or IL4I1 expression in OCCC patients, tumour rupture or chemoresistance. In contrast, bilateral tumours, lymph node metastasis, advanced stage, distant metastasis and abnormal cytology were significant risk factors negatively correlated with OCCC patient outcome (*p* < 0.05), with odds ratios (ORs) of 7.6, 5.5, 4.6, 4.3 and 2.7 respectively. Furthermore, lack of residual tumour after the first surgery was a protective factor for prognosis. However, only bilateral tumours and lymph node metastasis were shown to be significant risk factors related to OCCC prognosis in the multivariate statistical analysis (*p* < 0.01), with ORs of 4.4 and 3.7 respectively.

**TABLE 4:** Prognostic value of clinicopathological parameters in OCCC (PFS, *n* = 96).^a^

	Univariate				Multivariate			
	OR	95% CI		*p*	OR	95% CI		*p*
		Lower limit	Upper limit			Lower limit	Upper limit	
IDO1	1.763	0.611	5.082	0.294				
IDO2	25.212	0.034	1.873E+04	0.339				
TDO2	0.454	0.169	1.220	0.117				
IL4I1	1.190	0.337	4.194	0.787				
Late stage (III/IV)	4.299	1.695	11.589	**0.004** *	/	/	/	0.602
Bilateral tumours	7.600	2.824	20.458	**<0.001** *	4.446	1.772	11.157	**0.001** *
Lymph node metastasis	5.469	1.970	15.186	**0.001** *	3.714	1.382	9.982	**0.009** *
Distant metastasis	4.582	1.287	16.309	**0.019** *	/	/	/	0.404
Abnormal cytology	2.700	1.190	6.126	**0.017** *	/	/	/	0.362
Tumour rupture	0.890	0.330	2.400	0.818				
No residual tumour/R0	0.217	0.062	0.757	**0.017** *	/	/	/	0.836
Chemoresistance	8.069E+05	0.000	6.363E+76	0.870				

* *p* < 0.05 as significance.

^a^ Progression‐free survival (PFS) was used in univariate and multivariate prognostic analysis. Some cases were not involved in prognostic analysis because of incomplete medical records.

In addition, 96 OCCC patients were grouped into resistant and sensitive groups based on sensitivity to different platinum agents to assess the distribution of Trp catabolising enzyme expression. As shown in Table S2, we examined the relationship between chemosensitivity and the expression of the four proteins. We found that more than half of the OCCC samples in both the platinum‐resistant and platinum‐sensitive group expressed IDO2, TDO2 and IL4I1. Although the overall IDO1 positive expression rate was low in platinum‐resistant samples, they showed a trend for being more likely to be IDO1 positive (*p* = 0.056).

## 4 DISCUSSION

Our present study was the first to evaluate the expression of the Trp enzymes IDO1, IDO2, TDO2 and IL4I1 in OCCC, and the positive expression rates were considerably high, indicating a possible benefit of Trp enzyme‐targeted therapy in OCCC. More than 75% of samples stained positively for IDO2, TDO2 and IL4I1 on IHC, while approximately 26.8% of OCCC samples were IDO1 positive. Chemoresistant cases were more likely to be IDO1‐positive than platinum‐sensitive cases (46.7% vs. 19.8%). Furthermore, higher IDO1 expression was significantly correlated with advanced tumour stage and metastasis (Table 3). Consistently, positive IDO1 expression was associated with advanced stage in both ovarian serous carcinoma^32^, ^41^ and OCCC. Considering that the failure of IDO1 inhibitors in clinical trials might be a result of compensation by IDO2 or TDO,^26^ IDO1/IDO2 or IDO1/TDO dual‐target inhibitors might be better choices for the treatment of OCCC.

Correlation analysis showed that the OCCC cells that were IL4I1 positive were mainly IDO1 positive, while the majority of OCCC cells were positive for both TDO2 and IL4I1 (Table 2). To our knowledge, this is the first report of such relationships in OCCC tissues. Previously, it was shown that l‐tryptophan is a major substrate of both IDO1 and IL4I1, and high levels of its metabolites were associated with IL4I1 activity in ascites from HGSOC patients.^33^ Compared with that in normal tissue, TDO2 expression was upregulated in ovarian cancer tissues.^32^ The same was found to be true for IL4I1, a high level of which promoted ovarian progression.^42^ In a study of Merkel cell carcinoma (MCC), MCC cells with lower IDO1 expression but a tumour microenvironment with lower TDO2 and AhR expression had a longer overall survival rate.^43^ To target the IDO1/TDO2‐AhR pathway, IDO1, TDO2 and AhR expression should be investigated in cancer patients who plan to receive immunotherapy.

We also analysed independent risk factors related to OCCC outcomes, including IDO1, IDO2, TDO2 and IL4I1 expression and clinical features. According to previous studies, advanced stage, metastasis, bilateral tumours and tumour rupture were risk factors related to poor prognosis in ovarian cancers.^7^, ^44^, ^45^, ^46^, ^47^ In conclusion, positive IDO1, TDO2 and IL4I1 expression was considered as unfavourable factor of OCCC. Unfortunately, in our prognostic analysis, IDO1, IDO2, TDO2 and IL4I1 expression was not significantly associated with the OCCC survival rate, as shown in Table 4. Among the four clinical characteristics related to OCCC outcome, only bilateral tumours and lymph node metastasis of OCCC remained independent risk factors related to prognosis (Table 4). Previous studies showed that higher IDO1 expression in high‐grade ovarian adenocarcinoma is associated with better prognosis, including better overall and progression‐free survival rates.^48^, ^49^ In contrast, one study of HGSOC patients found that IDO1 expression was higher in platinum‐resistant patients than in platinum‐sensitive patients, and higher IDO1 expression was correlated with unfavourable prognosis.^41^ According to an analysis of gene expression data from The Cancer Genome Atlas and Genotype‐Tissue Expression project, the mRNA level of TDO2 is upregulated in ovarian serous cystadenocarcinoma in comparison to normal tissue, and higher TDO2 expression resulted in cancer progression.^32^ In a single‐cell study of IL4I1 in serous ovarian cancer, Kaplan–Meier curve analysis showed that higher IL4I1 expression was associated with poor OS.^42^

Certainly, our observational study has some limitations. For instance, our work lacks data from functional experiments or clinical trials to verify Trp metabolism enzyme expression in OCCC. The IDO1 expression pattern in our OCCC cohort was different from that in ovarian serous carcinoma, previously reported to range from 59% to 71%.^32^, ^41^ Efforts should be made to validate our findings.

## 5 CONCLUSION

In conclusion, our study might be the first to demonstrate the expression of four Trp metabolism enzymes (i.e. IDO1, IDO2, TDO2 and IL4I1) in OCCC tissues and their relationships with each other and clinical features. Most OCCC samples expressed IDO2, TDO2 and IL4I1. The expression of IDO1, IDO2, TDO2 and IL4I1 was related to different clinical characteristics. In terms of clinical characteristics, bilateral tumours were a significant risk factor predicting poor prognosis. These findings have the potential to optimise personalised therapies for OCCC patients.

## AUTHOR CONTRIBUTIONS

**Sumei zhang:** Conceptualization (equal); data curation (equal); formal analysis (equal); investigation (equal); methodology (equal); writing – original draft (equal); writing – review and editing (equal). **Yike Gao:** Conceptualization (equal); data curation (equal); formal analysis (equal); investigation (equal); methodology (equal); writing – original draft (equal); writing – review and editing (equal). **Pan Wang:** Formal analysis (equal); investigation (equal); methodology (equal). **Shu Wang:** Formal analysis (equal); investigation (equal); methodology (equal). **Yuming Wang:** Investigation (supporting); methodology (supporting). **Mei Li:** Investigation (supporting); methodology (supporting). **Anqi Wang:** Investigation (supporting); methodology (supporting). **Kun Zhao:** Investigation (supporting); methodology (supporting). **Zixin Zhang:** Investigation (supporting); methodology (supporting). **Jian Sun:** Conceptualization (equal); data curation (equal); formal analysis (equal); funding acquisition (equal); project administration (equal); supervision (equal); writing – review and editing (supporting). **Dan Guo:** Funding acquisition (equal); supervision (equal). **Zhiyong Liang:** Supervision (equal).

## FUNDING INFORMATION

This study is supported by the National High Level Hospital Clinical Research Funding 2023‐PUMCH‐F‐004 (D.G.), 2022‐PUMCH‐B‐062 (J.S.), 2022‐PUMCH‐D‐002 (D.G) and CAMS Innovation Fund for Medical Sciences (CIFMS) 2021‐I2M‐1‐053 (D.G.& Z.‐Y.L.).

## CONFLICT OF INTEREST STATEMENT

The authors declare no conflicts of interest.

## ETHICS STATEMENT

This study was performed in accordance with the Declaration of Helsinki and was approved by the Ethics Committee Review Board of Peking Union Medical College Hospital, China (ethics number: I‐22PJ112). Informed consent statement: Each patient/control individual provided written informed consent.

## Supporting information


Figure S1.

Table S1.
Table S2.

## Data Availability

The data are not publicly available due to ethical restrictions.
