# Supplementary figures and images for: Tryptophan metabolism enzymes are potential targets in ovarian clear cell carcinoma
Source: Cancer Med. 2023 Dec 7;12(24):21996–2005. doi: 10.1002/cam4.6778 (PMC10757115; doi:10.1002/cam4.6778)

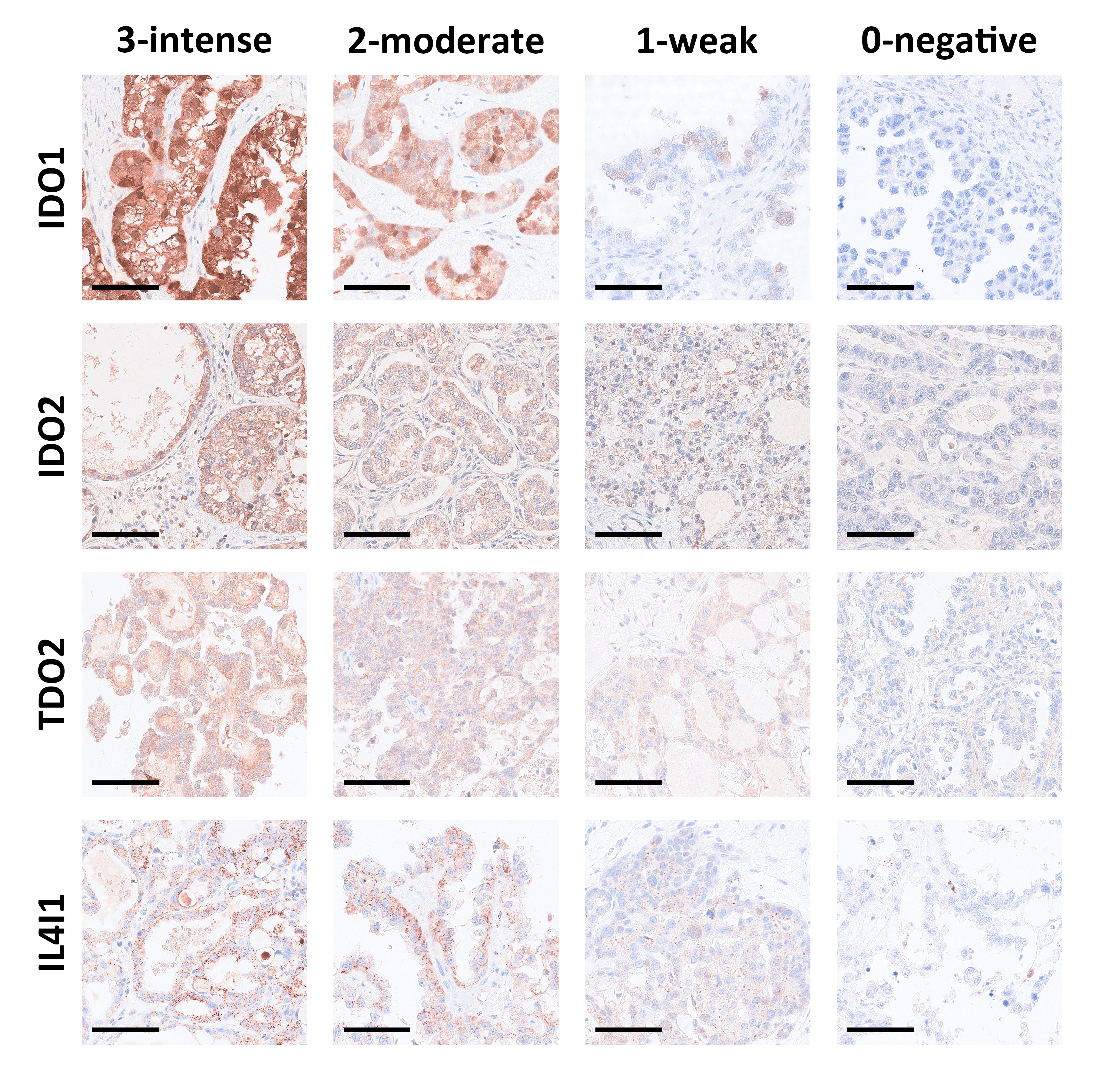

Supplement: Supplementary file 1 — Figure S1. [file CAM4-12-21996-s002.tif]
