# Supplementary material for: Tryptophan metabolism enzymes are potential targets in ovarian clear cell carcinoma
Source: Cancer Med. 2023 Dec 7;12(24):21996–2005. doi: 10.1002/cam4.6778 (PMC10757115; doi:10.1002/cam4.6778)
Supplement: Supplementary file 2 — Table S1. Table S2. [file CAM4-12-21996-s001.docx]

**Supplementary tables**

**Table S1 Expression and immunohistochemical control of the four antibodies**

|  | **Staining pattern** | **Positive control** | **Negative control** |
| --- | --- | --- | --- |
| **IDO1** | diffused staining in cytoplasm | endometrium carcinoma | internal control: stromal cells (fibroblast, etc) |
| **IDO2** | diffused staining in cytoplasm | normal liver |  |
| **TDO2** | diffused staining in cytoplasm | hepatocellular carcinoma |  |
| **IL4I1** | Scattered or diffused staining in cytoplasm | diffuse large B-cell lymphoma |  |

**Table S2 Expression of Trp catabolizing enzymes in patients with different platinum sensitivity (n=96)**

|  | Platinum-resistant (n=15) | Platinum-sensitive (n=81) | P value |
| --- | --- | --- | --- |
| IDO1 | 7 (46.7%) | 16 (19.8%) | 0.056 |
| IDO2 | 15 (100.0%) | 74 (91.4%) | 0.521 |
| TDO2 | 10 (66.7%) | 58 (71.6%) | 0.938 |
| IL4I1 | 12 (80.0%) | 66 (81.5%) | 1.000 |
